# Secondary primary malignancy in patients with head and neck squamous cell carcinoma: 27-year experience from the perspective of diagnostic tools

**DOI:** 10.1371/journal.pone.0263773

**Published:** 2022-02-15

**Authors:** Shih-Wei Wang, Leong-Perng Chan, Ling-Feng Wang, Che-Wei Wu, Sheng-Hsuan Lin, Tzu-Yen Huang, Ka-Wo Lee

**Affiliations:** 1 Department of Otolaryngology-Head and Neck Surgery, Kaohsiung Medical University Hospital, Kaohsiung, Taiwan; 2 Department of Otolaryngology-Head and Neck Surgery, Faculty of Medicine, College of Medicine, Kaohsiung Medical University, Kaohsiung, Taiwan; 3 Department of Otolaryngology-Head and Neck Surgery, Kaohsiung Municipal Tatung Hospital, Kaohsiung, Taiwan; 4 Department of Otolaryngology-Head and Neck Surgery, Kaohsiung Municipal Siaogang Hospital, Kaohsiung, Taiwan; 5 Institute of Data Science and Engineering, National Chiao Tung University, Hsinchu, Taiwan; 6 Department of Biological Science and Technology, National Chiao Tung University, Hsinchu, Taiwan; Istituto Nazionale Tumori IRCCS Fondazione Pascale, ITALY

## Abstract

**Background:**

The survival rate of head and neck squamous cell carcinoma (HNSCC) patients with secondary primary malignancy (SPM) showed no significant improvement for decades, however, the impact of advances in diagnostic tools is rarely mentioned. This study investigated the clinical characteristic of HNSCC with SPM over a 27-year period especially from the perspective of diagnostic tools.

**Methods:**

This study evaluated 157 HNSCC patients with SPM. The patients were divided into two groups according to the time of SPM diagnosis (Group A:1992–2003; Group B: 2004–2014). Age, gender, stage of first primary malignancy (FPM), SPM interval, overall survival, and disease-free survival were compared between groups.

**Results:**

Group B had significantly more SPM developed rate (p = 0.002), more SPM patients with advanced stage of FPM (p = 0.001), synchronous SPM (p = 0.006), and shorter SPM interval (p<0.001) compared to Group A. The survival rate in Group B was not significantly better than Group A.

**Conclusion:**

Among patients diagnosed with HNSCC recently, more SPMs are diagnosed in a shorter time interval and in a more advanced stage. The overall advances in diagnostic tools cannot significantly improve SPM survival, however, it enables more patients to receive corresponding treatment.

## Introduction

Head and neck squamous cell carcinoma (HNSCC) is the ninth most common malignancy in the world [1]. Patients with HNSCC have a high risk of second primary malignancy (SPM). SPM was defined by Warren and Gates in 1932 [2] and modified by Hong in 1990 [3]: 1) exclusion of the possibility that a tumor is a metastasis of another, 2) the time interval between SPM diagnosed at the same or an adjacent anatomical site of FPM should be at least 3 years 3) within three years of FPM diagnosis, SPM of the same histologic type as the FPM should be separated from the FPM by more than 2 cm of normal epithelium. The most common SPM sites are the head and neck region, the esophagus and the lungs [4–6]. An SPM is also an important negative prognostic factor in cancer survivors, which may lead to a decrease in the survival rate of HNSCC patients, one-third of deaths in HNSCC patients are attributable to SPM [7–9]. An international epidemiologic analysis reported that the cumulative incidence of SPM in HNSCC patients can reach 36% during the 20-year follow-up period [10].

The “field cancerization” hypothesis proposed by Slaughter et al. in 1953 provides a biological explanation of the occurrence of SPM [11]. That is, environmental carcinogens such as tobacco, alcohol and betel quid may induce field carcinogenesis, cause precancerous diseases, and increase epithelial cancer risk throughout the upper aerodigestive tract [12]. Other possible causes of SPM proposed in recent years include shared environmental or genetic risks and previous HNSCC treatment [13].

Although the modern treatment modalities do not significantly improve outcomes in the literature, the potentially large impact of advances in diagnostic tools has not been discussed extensively. To address this issue, this study analyzed 932 HNSCC cases treated and followed up by a single surgeon over a 27-year period in a medical center located in area of south Taiwan known to have a high incidence of HNSCC. The HNSCC cases that developed SPM were categorized into two groups (Group A:1992–2003; Group B: 2004–2014) according to the time of SPM diagnosis. The aim of this study was to compare the characteristics related to the advances in diagnostic tools, for example, diagnosis interval, cancer stage, patterns of FPM/SPM and survival rate, in two different time periods.

## Materials and methods

**Fig 1:**
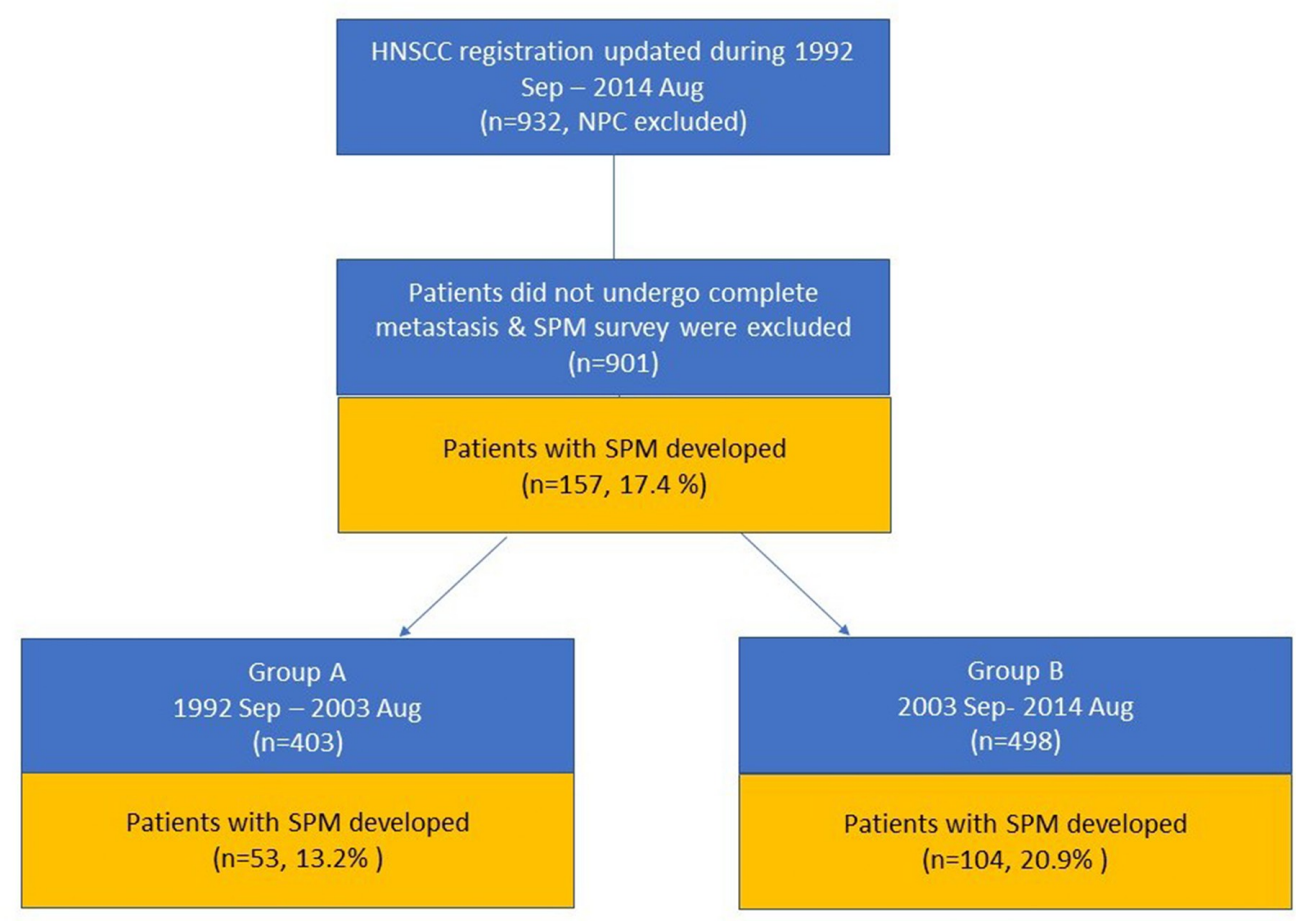
Flowchart of inclusion and exclusion procedure in each group.

This retrospective study enrolled 932 consecutive patients who had primary HNSCC originating in the oral cavity, oropharynx, larynx and hypopharynx diagnosed by a single surgeon (K-W, Lee) at Kaohsiung Medical University Hospital, Taiwan, from September, 1992, to August, 2014, and who had updated cancer registry information. As in previous studies of SPM, patients who had nasopharyngeal carcinoma (NPC) were excluded because of its distinct biologic behaviors and SPM patterns [14, 15]. In all HNSCC patients, organs that were the main sites of metastasis were regularly surveyed for 3 months after update of the cancer registry information, and all surveys were performed at least once annually thereafter. Routine surveys for metastasis and SPM for HNSCC patients included chest X-ray, abdominal sonography, esophagogastroduodenoscopy (EGD), and bone scintigraphy. Adjunct diagnostic tools including chest and abdominal computer tomography (CT) scans, and whole-body ^18^F-Fluorodeoxyglucose positron emission tomography (FDG-PET) scans were performed as needed, besides, FDG-PET had been available in our institution since Aug 2005. After excluding patients who did not undergo a complete survey, the analysis included 901 patients. Of these patients, 157 (17.4%) had developed SPM confirmed by pathology report. The SPM interval was defined as the time from the primary HNSCC diagnosis to the time of SPM diagnosis. All of the 157 patients treated in our institution had received SPM treatment and follow up. Follow up continued until loss of contact with the patient or until death. The last patient included in the analysis completed 5 years of follow up on August, 2019. Since September 2003, the institution had full adoption of the TCR (Taiwan Cancer Registry) reporting criteria, and a multidisciplinary committee was established. Fig 1 shows that the 157 SPM patients were also categorized into two groups according to time of SPM diagnosis: Group A included patients diagnosed from September, 1992, to August, 2003, and Group B included those diagnosed September, 2003, to August, 2014. Patient data collected in this study included age, gender, FPM subsites, and SPM sites. All methods were carried out in accordance with relevant guidelines and regulations. Ethical approval of this study was obtained from the Kaohsiung Medical University Hospital Institutional Review Board (KMUHIRB-E(I)-20200083).

This study defined SPM according to criteria proposed by Warren and Gates in 1932 [2] and modified by Hong in 1990 [3] mentioned in introduction section. A SPM that developed more than 6 months after an FPM diagnosis was classified as "metachronous"; one that developed within 6 months or less was classified as "synchronous" [2].

Staging was performed is according to American Joint Committee on Cancer (AJCC) guidelines. The 5th edition was used during 1997 to 2003, the 6th edition was used during 2003 to 2009, and the 7th edition was used during 2010 to 2017. Overall survival (OS) was defined as the length of time that the patient survived after the date of SPM diagnosis. Disease-free survival (DFS) was defined as the length of time that the patient survived without cancer signs or symptoms after the end of treatment for SPM.

To analyze the variables, independent t test and Pearson chi-square test were performed using SPSS (Version 18.0 for windows; SPSS Inc., Chicago, IL, USA). The Kaplan–Meier estimator, a standard non-parametric statistic used to estimate survival function of time-to-event data, was applied to measure the survival rate. A log-rank test was used to examine the difference in survival curves among different subpopulations. Both survival analyses were performed using R software (version-3.4). A two-tailed p value less than 0.05 was considered statistically significant.

## Results

Table 1 presents the characteristics of the patients. The 157 HNSCC patients with SPM included 152 (96.8%) men and 5 (3.2%) women with a mean age of 55.8 ± 9.7 years. According to AJCC criteria, 51, 32, 37 and 37 patients were in stages I, II, III and IV, respectively, at the time of primary HNSCC diagnosis. The average SPM interval was 46.5 ± 43.5 months. The mean 5-year OS and DFS rates were 39.6% and 35.4%, respectively.

**Table 1:** Demographic and clinical characteristics in HNSCC patients of Group A and Group B.

	Total	Group A	Group B	p value
SPM developed (%)	157/901 (17.4)	53/403 (13.2)	104/498 (20.9)	0.002
Age^a^ (mean±SD)	55.8 ± 9.7	54.6 ± 9.5	56.3 ± 9.7	0.281
Gender (Male/Female)	152/5	53/0	99/5	0.254
Subsite of FPM^b^				
Oral cavity (%)	53/901 (5.9)	23/403 (5.7)	30/498 (6.0)	0.841
Oropharynx (%)	31/901 (3.4)	8/403 (2.0)	23/498 (4.6)	0.031
Larynx (%)	32/901 (3.6)	12/403 (3.0)	20/498 (4.0)	0.402
Hypopharynx (%)	40/901 (4.4)	9/403 (2.2)	31/498 (6.2)	0.004
Stage of FPM^c^				0.001
Early stages I / II	51 / 32	22 / 16	29 / 16	
Advanced stages III / IV	37 / 37	7 / 8	30 / 29	
Site of SPM^d^				
Head and Neck (%)	86/901 (9.5)	36/403 (8.9)	50/498 (10.0)	0.574
Esophagus (%)	26/901 (2.9)	5/403 (1.2)	21/498 (4.2)	0.008
Liver (%)	10/901 (1.1)	1/403 (0.2)	9/498 (1.8)	0.057
Lung (%)	8/901 (0.9)	0/403 (0.0)	8/498 (1.6)	0.028
Colon (%)	8/901 (0.9)	3/403 (0.7)	5/498 (1.0)	0.955
SPM interval^e^ (months, mean±SD)	46.5 ± 43.5	72.8 ± 49.0	33.2 ± 33.4	<0.001
SPM interval type				0.006
Synchronous (%)	31 (19.7)	4 (7.5)	27 (26.0)	
Metachronous (%)	126 (80.3)	49 (92.5)	77 (74.0)	
Five-year OS rate (%)	57/144 (39.6)	19/51 (37.3)	38/93 (40.9)	0.674
Five-year DFS rate (%)	51/144 (35.4)	17/51 (33.3)	34/93 (36.6)	0.699
Survey for SPMs				
PET-CT (%)	20/157 (12.7)	0/53 (0.0)	20/104 (19.2)	
Serial surveys	137 patients	53 patients	84 patients	
CXR or Chest CT	137/137(100.0)	53/53(100.0)	84/84(100.0)	
Abd echo	129/137(94.2)	48/53(90.1)	81/84(96.4)	
Bone scan	126/137(92.0)	46/53(86.8)	80/84(95.2)	
EGD	81/137(59.1)	17/53 (32.1)	64/84 (76.2)	
Treatment				0.008
OP (-ND)	24 (15.3)	11 (20.8)	13 (12.5)	
OP (+ND)	31 (19.7)	15 (28.3)	16 (15.4)	
OP + RT^f^	34 (21.7)	14 (26.4)	20 (19.2)	
OP + CCRT^g^	19 (12.1)	6 (11.3)	13 (12.5)	
CCRT^g^	49 (31.2)	7 (13.2)	42 (40.4)	

HNSCC = head and neck squamous cell carcinoma; FPM = first primary malignancy; SPM = second primary malignancy; SD = standard deviation; OS = overall survival; DFS = disease free survival; OP = operation; ND = neck dissection; RT = radiotherapy; CCRT = concurrent chemoradiotherapy; PET-CT = Positron emission tomography-Computed tomography; CXR = Chest X-ray; Chest CT = Chest computed tomography; Abd echo = Abdominal echography; EGD = esophagogastroduodenoscopy

^a^ Age at diagnosis of SPM

^b^ One patient in Group A had FPM located in nasal cavity

^c^ Staging according to the version of American Joint Committee on Cancer (AJCC) guidelines at the time of diagnosis

^d^ 2, 3, 2, and 1 patients in Group A developed SPM in the stomach, nasopharynx, bladder, and prostate, respectively; 4, 1, 2, 1, 1, 1, and 1 patients in Group B developed SPM in the stomach, nasopharynx, bladder, prostate, parotid gland, pancreas, and thyroid, respectively.

^e^ Time interval from FPM diagnosis to SPM diagnosis

^f^ The dose of RT was 66–70 Gy

^g^ The dose of cisplatin was applied mostly 75 mg/m^2^ every 3 weeks (2–3 cycles) or 30 mg/m^2^ weekly (6–8 weeks), followed by concurrent RT (dose: 66–70 Gy).

The patients were then subdivided according to the time of SPM diagnosis: 53 (13.2%) of 403 patients in Group A, and 104 (20.9%) of 498 patients in Group B. The proportion of patients diagnosed with SPM was significantly larger in Group B compared to Group A (p = 0.002), but age and gender did not significantly differ.

Comparison of FPM subsites between groups, in each subsite, the percentage of patients with FPM was significantly larger in Group B compared to Group A. Group B also had significantly more FPMs located in the oropharynx (p = 0.031) and in the hypopharynx (p = 0.004) compared to Group A.

Comparison of FPM stages between groups, Group A also had significantly more cases in early FPM stages (I and II) compared to Group B whereas Group B had significantly more cases in advanced FPM stages (III and IV) compared to Group A (p = 0.001).

Comparison of SPM sites between groups, Group B had a larger percentage of patients with SPM in each site compared to Group A. The difference was statistically significant in patients who had SPM in the esophagus (p = 0.008) and in the lungs (p = 0.028).

The SPM interval was significantly (p<0.001) longer in Group A (72.8 ± 49.0 months) than in Group B (33.2 ± 33.4 months). Group A had 4 synchronous and 49 metachronous SPMs whereas Group B had 27 synchronous and 77 metachronous SPMs. Thus, Group B had significantly (p = 0.006) more proportion of synchronous SPMs compared to Group A. The 5-year OS rate did not significantly (p = 0.674) differ between Group A (37.3%) and Group B (40.9%). Additionally, the 5-year DFS rate did not significantly (p = 0.699) differ between Group A (33.3%) and Group B (36.6%).

The treatment of HNSCC patients showed significant difference (p = 0.008) between groups, especially in the patients received CCRT (13.2% in Group A vs. 40.4% in Group B).

**Fig 2:**
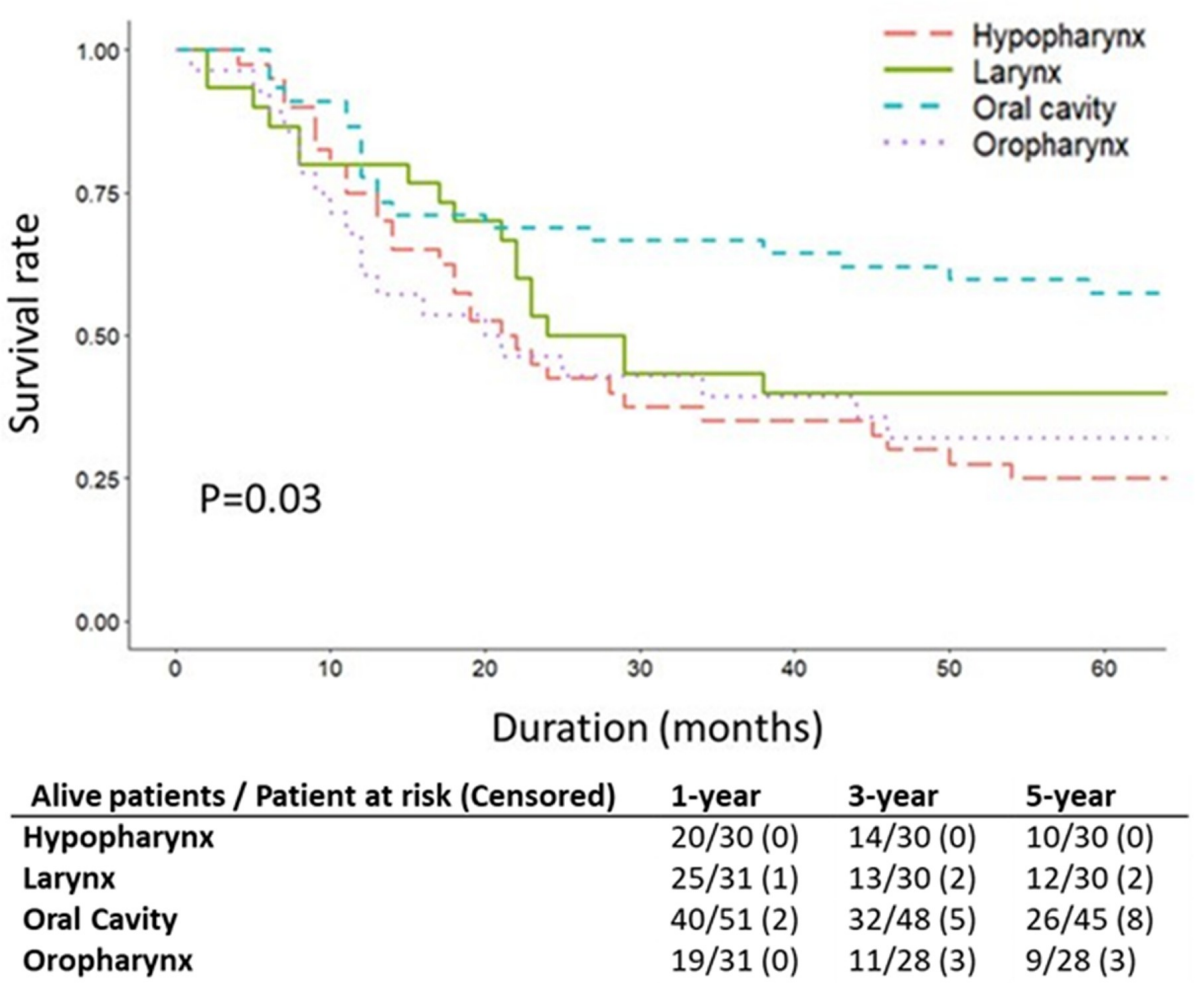
Kaplan-Meier survival curves for first primary malignancy subsites in cases who developed secondary primary malignancy.

Fig 2 shows the 5-year OS rates for each FPM subsite. The 5-year OS rates significantly (p = 0.03) differed in each subsite: 57.8% for oral cavity, 32.1% for oropharynx, 40.0% for larynx, and 25.0% for hypopharynx.

**Fig 3:**
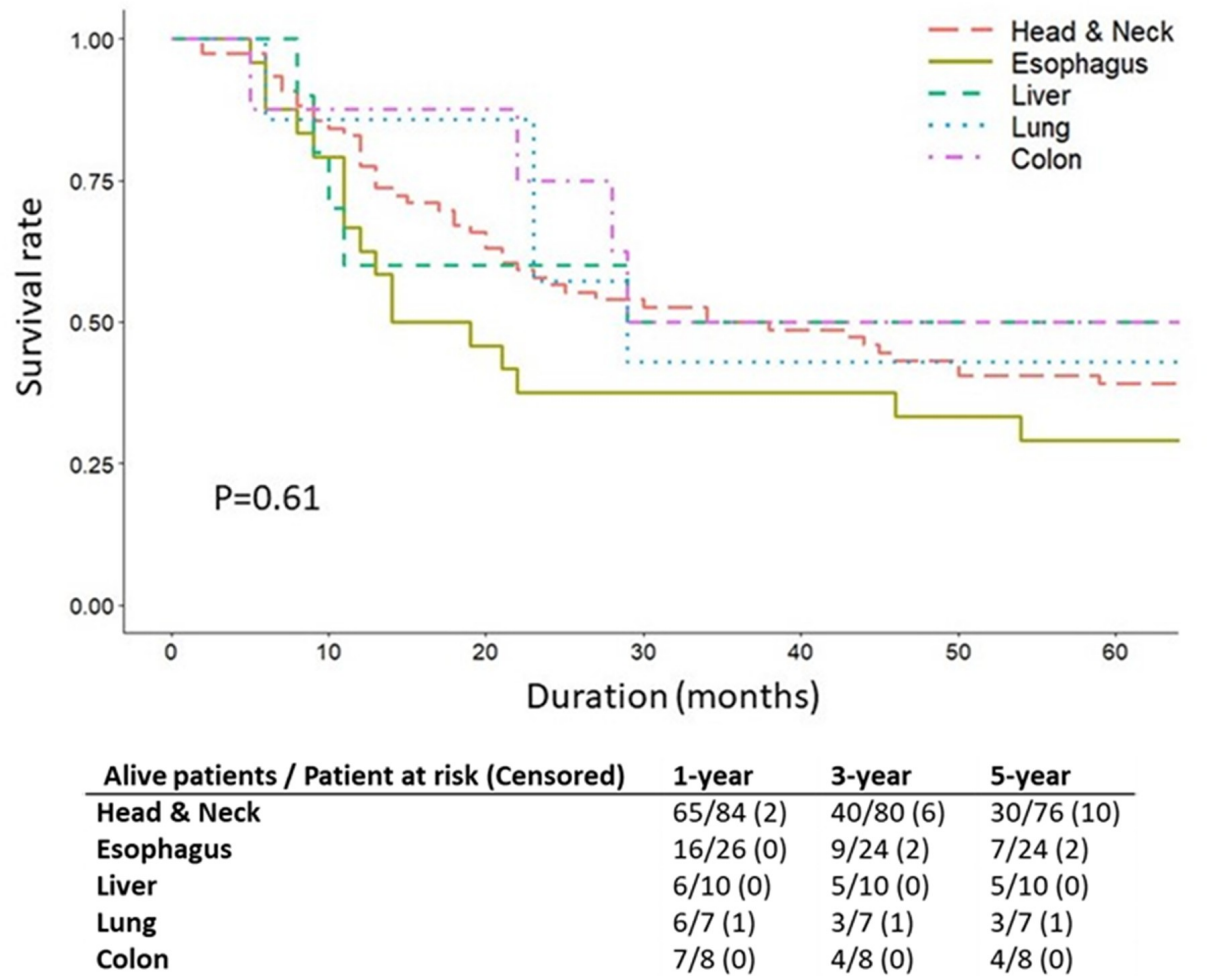
Kaplan-Meier survival curves for different second primary malignancy (SPM) sites in 157 SPM patients.

Fig 3 compares survival by SPM sites. The 5-year OS rates for SPM in the head and neck region, esophagus, liver, lung and colon, were 39.5%, 29.2%, 50.0%, 42.9% and 50.0%, respectively. Comparison of data for all SPM sites revealed that OS was lowest for SPM in the esophagus, but the difference did not reach statistical significance (p = 0.61).

**Fig 4:**
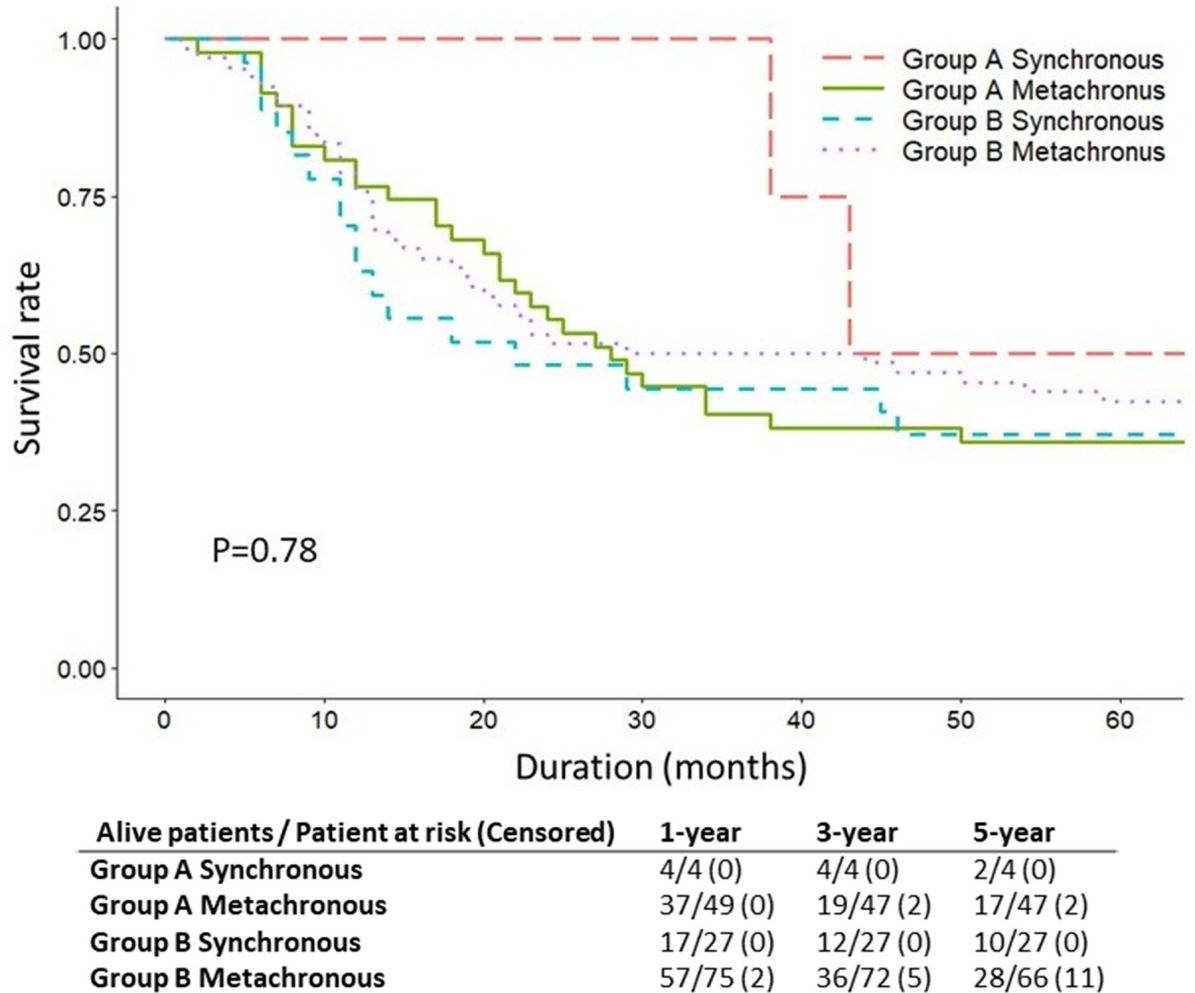
Kaplan-Meier survival curves for synchronous and metachronous second primary malignancy (SPM) in Group A and Group B.

In Fig 4, survival of patients with synchronous SPM and metachronous SPM is compared between Group A and Group B. In Group A, the 5-year OS was 50.0% and 36.2% in synchronous and metachronous SPM, respectively; in Group B, the 5-year OS was 37.0% and 42.4% synchronous and metachronous SPM, respectively. The differences in 5-year OS did not reach statistical significance (p = 0.78).

## Discussion

The HNSCC cases in this study were categorized into two groups according to time of SPM diagnosis. Compared to Group A, Group B had significantly more SPM developed rate (p = 0.002), more SPM patients with advanced stage of FPM (p = 0.001), synchronous SPM (p = 0.006), and shorter SPM interval (p<0.001). Compared to Group A, Group B had more FPMs in the oropharynx (p = 0.031) and in the hypopharynx (p = 0.004) and had more SPMs in the esophagus (p = 0.008) and in the lungs (p = 0.028). Finally, Group B had slightly better 5-year OS and DFS rates compared to Group A, but the differences were not statistically significant.

In recent decades, several major improvements in diagnostic tools have been widely accepted for clinical use. Examples include whole-body FDG-PET scan [16, 17] and lugol chromo-EGD combined with narrow band imaging (NBI) [18, 19]. For SPM out of the head and neck region, these imaging technologies offer more functional features for detecting SPM in the early stage; for SPM in the head and neck region, these images have advantages to differentiate SPM from post-treatment changes [20, 21]. For detecting lung metastases or lung SPMs, chest CT is more accurate than chest X-ray [22]. For maximum cost effectiveness and minimal radiation exposure, recent international guidelines recommend the use of regular low-dose lung CT scans for lung malignancy screening [23, 24].

Comparisons of SPM sites in each group in this study indicated that the improved accessibility and lower cost of chest CT scans may explain why the number of secondary primary lung cancer diagnoses was significantly (p = 0.028) larger in Group B compared to Group A. Improving sensitivity of abdominal sonography has also increased accuracy in detection of secondary primary liver cancer and further confirmation by abdominal CT or magnetic resonance imaging scans. However, the percentage increase in diagnoses of secondary primary esophageal cancer was significantly larger in Group B (p = 0.008) compared to other SPM sites. The “field cancerization” concept which emphasizes the relationship between HNSCC and esophageal cancer, has resulted in the preferential use of EGD for HNSCC survey, and the use of its adjuncts (i.e., lugol, NBI) has also increased [25]. This phenomenon may explain why the number of SPM diagnoses was significantly higher in patients with FPM in the oropharynx (p = 0.031) or in the hypopharynx (0.004) compared to patients with FPM in the larynx (p = 0.402). Local examinations for head and neck cancer are still mainly performed by visual inspection, palpation, fiberlaryngoscopy, and head and neck CT scan. Slow implementation of new optical technologies (i.e., NBI and optical coherence tomography) may explain why the rate of second primary head and neck cancer has not significantly increased. Finally, colonoscopy is not routinely used for HNSCC survey. Considering the annual increases in the prevalence of colorectal cancer, the existing data does not show a clear advantage of PET scan for SPM diagnosis.

Comparisons revealed that Group B had a significantly shorter SPM interval and significantly more synchronous SPM cases compared to Group A. Advances in diagnostic tools now enable more accurate detection of SPM at an earlier SPM stage, which might improve survival rates. However, some studies have reported that survival is poorer in synchronous SPM than in metachronous SPM [26–28]. A possible explanation for the poorer survival in synchronous SPM is that, because of the toxicity of FPM treatment and the decreased nutritional intake during FPM treatment, synchronous SPM cannot be treated as aggressively as metachronous SPM [29]. Additionally, the proportion of patients in this study with an advanced stage of FPM was larger in Group B compared to Group A, and Parry et al. [30] reported similar results. The increasing proportion of SPM patients with an advanced stage of FPM may negatively affect the survival rate. In this study, the 5-year OS did not significantly differ by synchronous or metachronous SPM in both groups (p = 0.78).

In this study, the proportion of synchronous SPM in Group B is significantly higher than Group A (p = 0.006). Among them, there are 10 cases of synchronous SPM in Group B that occurs in the esophagus, which is highly associated with the advancement of EGD (lugol and NBI). In order to analyze the impact of diagnostic tools during the follow-up period, Table 2 shows the characteristics in metachronous SPM of groups. In this table, there are still significant differences in stage of FPM (p = 0.004) and interval of SPM (78.7±46.2 months in Group A, 44.1±32.3 months in Group B, p<0.001). While survival improvement is still limited, indicating the advancement of overall diagnostic tools also plays an important role in detecting more SPM during the follow-up period of metachronous SPMs.

**Table 2:** Demographic and clinical characteristics in HNSCC patients with metachronous SPM of Group A and Group B.

	Metachronous SPM of Group A (N = 49)	Metachronous SPM of Group B (N = 77)	p value
Age^a^ (mean±SD)	54.2 ± 9.7	56.2 ± 10.4	NS
Gender (Male/Female)	49/0	72/5	NS
Subsite of FPM			NS
Oral cavity	22	25	
Oropharynx	6	20	
Larynx	11	14	
Hypopharynx	9	18	
Other	1	0	
Stage of FPM^c^			0.004
Early stages I / II	20 / 15	23 / 12	
Advanced stages III / IV	7 / 7	22 / 20	
Site of SPM			NS
Head and Neck	33	44	
Esophagus	5	11	
Liver	1	4	
Lung	0	5	
Colon	3	4	
Other	8	11	
SPM interval^c^ (months, mean±SD)	78.7 ± 46.2	44.1 ± 32.3	<0.001
Five-year OS rate (%)	17/47 (36.2)	28/66 (42.4)	NS
Five-year DFS rate (%)	15/47 (31.9)	26/66 (39.4)	NS

NS = not significant

^a^ Age at diagnosis of SPM

^b^ Staging according to the version of American Joint Committee on Cancer (AJCC) guidelines at the time of diagnosis

^c^ Time interval from FPM diagnosis to SPM diagnosis.

Studies suggest that a multidisciplinary approach to treatment and supportive care for HNSCC patients improves their life expectancy and quality of life as well as their awareness of the importance of early detection of the disease [31]. Our institution implemented a multidisciplinary approach to treating HNSCC in 2003 and established a multidisciplinary HNSCC treatment committee in 2008. However, the 5-year OS and DFS of SPM in Group B was not significantly better than those in Group A, which is consistent with the overall results of survival studies performed in different decades [32, 33].

The SPM survival rate is related to the prognostic characteristics of FPM. The results of this study indicate that the FPM subsite is a significant predictor of survival (p = 0.03). In the literature, whether the SPM site predicts survival is controversial. Some studies have reported that patients with SPM in the head and neck have a better prognosis compared to those with SPM in the lungs or esophagus [10, 26, 34]. In our study, patients with a secondary primary esophageal cancer had worse outcomes compared to patients with other SPM types, but the difference was not statistically significant (p = 0.61). Although SPM is a strong negative prognostic indicator in HNSCC, the survival impact of SPM site may not be as large as the survival impact of FPM site.

A superior diagnostic tool for SPM screening is required. Some universalized and readily accessible biomarker assessments have been developed, e.g., molecular analysis, markers such as loss of heterozygosity (LOH), microsatellite alterations, chromosomal instability, mutations in the TP53 gene, DNA amplification techniques and immunohistochemistry [35]. Targeted therapy, immunotherapy, cellular therapy and cancer gene therapy may have important roles in SPM treatment. Also the advanced computational imaging analysis and artificial intelligence application to predict treatment outcome in patients with HNSCC is an emerging field in oncology [36, 37]. Use of these therapies individually or in combination may offer advantages such as decreased toxicity, increased tolerance, and increased compliance. Further research is needed to develop novel diagnostic and treatment methods specifically for treating SPM.

This study had some limitations. First, the case number in this study was much smaller compared to that in the Taiwan Cancer Registry (TCR) or in other meta-analysis studies. However, the TCR does not contain complete and detailed staging and treatment data for FPM patients treated before year 2002. Since all SPM patients in our study were followed up by a single surgeon in a single medical center over a 27-year period, our study collected information that is unavailable in the TCR. Second, although this study excluded NPC, it did not exclude another virus-related HNSCC, which is an HPV-positive oropharyngeal cancer, because routine p16 testing for all oropharyngeal cancers has only been available in the past 3 years. This study did not investigate the impact of diagnosis and treatment of HPV-positive oropharyngeal cancer. Third, this study did not include patients with non-HNSCC as FPM and HNSCC as SPM. This study design may affect the SPM diagnosis and survival rates compared with other literature, however, the design is necessary to evaluate the differences resulted from advances in diagnostic tools of SPM between groups. Fourth, the impact of different treatment method, medical habits change and accessibility of examinations were difficult to quantitatively compared between groups. The consistent strategy of routine metastasis and SPM surveys cannot completely avoid this bias. Finally, changes in staging guidelines during this long-term 27-year study may have biased the results. Nevertheless, criteria for distinguishing early and advanced stage cancers have been relatively consistent during this period.

## Conclusion

Among patients diagnosed with HNSCC recently, more SPMs are diagnosed in a shorter time interval and in a more advanced stage. The overall advances in diagnostic tools cannot significantly improve SPM survival, however, it enables more patients to receive corresponding treatment. Further research in HNSCC patients with SPM is needed to establish better and more cost-effective diagnostic tools and treatment modalities.

## References

[1] Bray F, Ferlay J, Soerjomataram I, Siegel RL, Torre LA, Jemal A. Global cancer statistics 2018: GLOBOCAN estimates of incidence and mortality worldwide for 36 cancers in 185 countries. CA Cancer J Clin. 2018;68(6):394–424. doi: 10.3322/caac.21492 30207593

[2] Warren S. Multiple primary malignant tumors. A survey of the literature and a statistical study. Am J cancer 1932:1358–414.

[3] Hong WK, Lippman SM, Itri LM, Karp DD, Lee JS, Byers RM, et al. Prevention of second primary tumors with isotretinoin in squamous-cell carcinoma of the head and neck. N Engl J Med. 1990;323(12):795–801. doi: 10.1056/NEJM199009203231205 2202902

[4] Licciardello JT, Spitz MR, Hong WK. Multiple primary cancer in patients with cancer of the head and neck: second cancer of the head and neck, esophagus, and lung. Int J Radiat Oncol Biol Phys. 1989;17(3):467–76. doi: 10.1016/0360-3016(89)90096-5 2674075

[5] Priante AV, Castilho EC, Kowalski LP. Second primary tumors in patients with head and neck cancer. Curr Oncol Rep. 2011;13(2):132–7. doi: 10.1007/s11912-010-0147-7 21234721

[6] Morris LGT, Sikora AG, Hayes RB, Patel SG, Ganly I. Anatomic sites at elevated risk of second primary cancer after an index head and neck cancer. Cancer Causes & Control. 2011;22(5):671–9. doi: 10.1007/s10552-011-9739-2 21327458 PMC3085084

[7] Vikram B. Changing patterns of failure in advanced head and neck cancer. Arch Otolaryngol. 1984;110(9):564–5. doi: 10.1001/archotol.1984.00800350006003 6477272

[8] Sturgis EM, Miller RH. Second primary malignancies in the head and neck cancer patient. Ann Otol Rhinol Laryngol. 1995;104(12):946–54. doi: 10.1177/000348949510401206 7492066

[9] Le QC, Arimura H, Ninomiya K, Kabata Y. Radiomic features based on Hessian index for prediction of prognosis in head-and-neck cancer patients. Scientific reports. 2020;10(1):1–12. doi: 10.1038/s41598-019-56847-4 33277570 PMC7718925

[10] Chuang S-C, Scelo G, Tonita JM, Tamaro S, Jonasson JG, Kliewer EV, et al. Risk of second primary cancer among patients with head and neck cancers: A pooled analysis of 13 cancer registries. International Journal of Cancer. 2008;123(10):2390–6. doi: 10.1002/ijc.23798 18729183

[11] Slaughter DP, Southwick HW, Smejkal W. “Field cancerization” in oral stratified squamous epithelium. Clinical implications of multicentric origin. Cancer. 1953;6(5):963–8. doi: 10.1002/1097-0142(195309)6:5&lt;963::aid-cncr2820060515&gt;3.0.co;2-q 13094644

[12] Ha PK, Califano JA. The molecular biology of mucosal field cancerization of the head and neck. Crit Rev Oral Biol Med. 2003;14(5):363–9. doi: 10.1177/154411130301400506 14530304

[13] Braakhuis BJ, Tabor MP, Kummer JA, Leemans CR, Brakenhoff RH. A genetic explanation of Slaughter’s concept of field cancerization: evidence and clinical implications. Cancer Res. 2003;63(8):1727–30. 12702551

[14] Chien YC, Chen JY, Liu MY, Yang HI, Hsu MM, Chen CJ, et al. Serologic markers of Epstein-Barr virus infection and nasopharyngeal carcinoma in Taiwanese men. N Engl J Med. 2001;345(26):1877–82. doi: 10.1056/NEJMoa011610 11756578

[15] Chen MC, Feng IJ, Lu CH, Chen CC, Lin JT, Huang SH, et al. The incidence and risk of second primary cancers in patients with nasopharyngeal carcinoma: a population-based study in Taiwan over a 25-year period (1979–2003). Annals of Oncology. 2008;19(6):1180–6. doi: 10.1093/annonc/mdn003 18238781

[16] Goerres GW, von Schulthess GK, Steinert HC. Why most PET of lung and head-and-neck cancer will be PET/CT. The Journal of Nuclear Medicine. 2004;45:66S. 14736837

[17] Park J, Pak K, Yun TJ, Lee EK, Ryoo I, Lee JY, et al. Diagnostic Accuracy and Confidence of [18F] FDG PET/MRI in comparison with PET or MRI alone in Head and Neck Cancer. Scientific reports. 2020;10(1):1–8. doi: 10.1038/s41598-019-56847-4 32528161 PMC7289810

[18] Lee Y-C, Wang C-P, Chen C-C, Chiu H-M, Ko J-Y, Lou P-J, et al. Transnasal endoscopy with narrow-band imaging and Lugol staining to screen patients with head and neck cancer whose condition limits oral intubation with standard endoscope (with video). Gastrointestinal endoscopy. 2009;69(3):408–17. 19019362 10.1016/j.gie.2008.05.033

[19] Takenaka R, Kawahara Y, Okada H, Hori K, Inoue M, Kawano S, et al. Narrow-band imaging provides reliable screening for esophageal malignancy in patients with head and neck cancers. American Journal of Gastroenterology. 2009;104(12):2942–8. doi: 10.1038/ajg.2009.426 19623169

[20] Nevens D, Vantomme O, Laenen A, Hermans R, Nuyts S. CT-based follow-up following radiotherapy or radiochemotherapy for locally advanced head and neck cancer; outcome and development of a prognostic model for regional control. The British journal of radiology. 2016;89(1068):20160492. doi: 10.1259/bjr.20160492 27710014 PMC5604915

[21] Becker M, Varoquaux AD, Combescure C, Rager O, Pusztaszeri M, Burkhardt K, et al. Local recurrence of squamous cell carcinoma of the head and neck after radio (chemo) therapy: diagnostic performance of FDG-PET/MRI with diffusion-weighted sequences. European radiology. 2018;28(2):651–63. doi: 10.1007/s00330-017-4999-1 28812148 PMC5740208

[22] Rohde M, Nielsen AL, Johansen J, Sørensen JA, Nguyen N, Diaz A, et al. Head-to-head comparison of chest x-ray/head and neck MRI, chest CT/head and neck MRI, and 18F-FDG PET/CT for detection of distant metastases and synchronous cancer in oral, pharyngeal, and laryngeal cancer. Journal of Nuclear Medicine. 2017;58(12):1919–24. doi: 10.2967/jnumed.117.189704 28572489

[23] Griffioen GH, Louie AV, de Bree R, Smit EF, Paul MA, Slotman BJ, et al. Second primary lung cancers following a diagnosis of primary head and neck cancer. Lung cancer. 2015;88(1):94–9. doi: 10.1016/j.lungcan.2015.01.011 25662386

[24] Jacobson FL, Austin JH, Field JK, Jett JR, Keshavjee S, MacMahon H, et al. Development of The American Association for Thoracic Surgery guidelines for low-dose computed tomography scans to screen for lung cancer in North America: recommendations of The American Association for Thoracic Surgery Task Force for Lung Cancer Screening and Surveillance. The Journal of Thoracic and Cardiovascular Surgery. 2012;144(1):25–32. doi: 10.1016/j.jtcvs.2012.05.059 22710038

[25] Tseng C-M, Wang H-H, Lee C-T, Tai C-M, Tseng C-H, Chen C-C, et al. A nationwide population-based study to access the risk of metachronous esophageal cancers in head and neck cancer survivors. Scientific reports. 2020;10(1):1–7. doi: 10.1038/s41598-019-56847-4 31964952 PMC6972960

[26] Panosetti E, Luboinski B, Mamelle G, Richard JM. Multiple synchronous and metachronous cancers of the upper aerodigestive tract: a nine-year study. Laryngoscope. 1989;99(12):1267–73. doi: 10.1288/00005537-198912000-00011 2601541

[27] Lin K, Patel SG, Chu PY, Matsuo JM, Singh B, Wong RJ, et al. Second primary malignancy of the aerodigestive tract in patients treated for cancer of the oral cavity and larynx. Head Neck. 2005;27(12):1042–8. doi: 10.1002/hed.20272 16265657

[28] Di Martino E, Sellhaus B, Hausmann R, Minkenberg R, Lohmann M, Esthofen MW. Survival in second primary malignancies of patients with head and neck cancer. J Laryngol Otol. 2002;116(10):831–8. doi: 10.1258/00222150260293664 12437840

[29] León X, Ferlito A, III CMM, Saffiotti U, Shaha AR, Bradley PJ, et al. Second primary tumors in head and neck cancer patients. Acta oto-laryngologica. 2002;122(7):765–78. 12484655

[30] Parry C, Kent EE, Mariotto AB, Alfano CM, Rowland JH. Cancer survivors: a booming population. Cancer Epidemiology and Prevention Biomarkers. 2011;20(10):1996–2005. doi: 10.1158/1055-9965.EPI-11-0729 21980007 PMC3422885

[31] Nigro CL, Denaro N, Merlotti A, Merlano M. Head and neck cancer: improving outcomes with a multidisciplinary approach. Cancer management and research. 2017;9:363. doi: 10.2147/CMAR.S115761 28860859 PMC5571817

[32] Chen M-C, Huang W-C, Chan CH, Chen P-T, Lee K-D. Impact of second primary esophageal or lung cancer on survival of patients with head and neck cancer. Oral Oncology. 2010;46(4):249–54. doi: 10.1016/j.oraloncology.2010.01.002 20138797

[33] Rafferty M, O’dwyer T. Secondary primary malignancies in head and neck squamous cell carcinoma. The Journal of Laryngology and Otology. 2001;115(12):988. doi: 10.1258/0022215011909567 11779329

[34] Tsou YA, Hua CH, Tseng HC, Lin MH, Tsai MH. Survival study and treatment strategy for second primary malignancies in patients with head and neck squamous cell carcinoma and nasopharyngeal carcinoma. Acta Otolaryngol. 2007;127(6):651–7. doi: 10.1080/00016480600951517 17503236

[35] Cui J, Zheng L, Zhang Y, Xue M. Bioinformatics analysis of DNMT1 expression and its role in head and neck squamous cell carcinoma prognosis. Scientific reports. 2021;11(1):1–11. doi: 10.1038/s41598-020-79139-8 33500531 PMC7838186

[36] Fontaine P, Acosta O, Castelli J, De Crevoisier R, Müller H, Depeursinge A. The importance of feature aggregation in radiomics: a head and neck cancer study. Scientific Reports. 2020;10(1):1–11. doi: 10.1038/s41598-019-56847-4 33184313 PMC7661538

[37] Bogowicz M, Tanadini-Lang S, Guckenberger M, Riesterer O. Combined CT radiomics of primary tumor and metastatic lymph nodes improves prediction of loco-regional control in head and neck cancer. Scientific reports. 2019;9(1):1–7. doi: 10.1038/s41598-018-37186-2 31645603 PMC6811564

